# “Covid‐19 is dangerous”: The role of parental verbal threat information on children's fear of Covid‐19

**DOI:** 10.1002/jad.12105

**Published:** 2022-10-25

**Authors:** Cosima A. Nimphy, Bernet M. Elzinga, Willem Van der Does, Evin Aktar

**Affiliations:** ^1^ Department of Clinical Psychology Leiden University Leiden The Netherlands; ^2^ Leiden Institute for Brain and Cognition (LIBC) Leiden The Netherlands

**Keywords:** child temperament, Covid‐19 fear, information transmission, parental anxiety, parental negative comments

## Abstract

**Introduction:**

Theoretical and empirical evidence suggests that the effect of parental verbal threat information on the offspring's fear acquisition of novel stimuli may be causal. The current study investigated this verbal fear acquisition pathway from parents to children in the unique context of Covid‐19 as a novel environmental threat for parents and children.

**Methods:**

Using an online cross‐sectional survey, we collected data about fear of Covid‐19, parent–child communication, parental anxiety, and child temperament, in the period between June 11th 2020 and May 28th 2021. Participants were 8 to 18‐year‐old children (*N* = 195; *M*_age_ = 14.23; 113 girls) and their parents (*N* = 193; *M*_age_ = 47.82; 146 mothers) living in the Netherlands.

**Results:**

Children of parents with stronger Covid‐19 fears also reported stronger Covid‐19 fears. Moreover, parents who were more fearful of Covid‐19 provided more threat‐related information about the virus to their children. More parental threat information in turn was related to stronger fear of Covid‐19 in their children, and partly mediated the link between parent and child fear of the virus. The link between parental threat information and children's fear of Covid‐19 was not moderated by child temperament or parental anxiety.

**Conclusions:**

Parental communication about Covid‐19 may play a role in children's fear acquisition of Covid‐19. The lack of moderation of this link by parental anxiety and child temperament may reflect the potentially adaptive nature of verbal fear transmission during the first year of the pandemic and the nonclinical levels of fear in this community sample.

## 1 INTRODUCTION

The Covid‐19 pandemic has led many to worry about their own physical and mental health, as well as of significant others (Taylor et al., 2020). While fear is a natural and adaptive response to potentially threatening situations, it can become maladaptive when it is disproportional to the severity of the threat and impairs daily functioning (Delgado et al., 2006; Ferrari, 1986). Individuals suffering from Covid‐19 anxiety report lower well‐being, increased psychological distress, and safety behaviors, such as hand washing and social isolation (Chen et al., 2021; Knowles & Olatunji, 2021). Yet, more research is needed to understand how adaptive fear of Covid‐19 develops, before investigating the development and prevention of Covid‐19 anxiety.

The development of Covid‐19 fears can be conceptualized within the broader context of fear‐acquisition frameworks and social fear learning. One of the ways that fears may be acquired is via direct aversive experiences (classical conditioning; Pavlov, 1927). For instance, people may learn to fear a situation (such as a crowded area due to risk of contamination) following an aversive experience with Covid‐19, for example in cases where they got severely sick or lost a loved one. However, personal experiences are not even necessary, since it is possible to acquire fears indirectly from others (social fear learning; Olsson et al., 2007; Rachman, 1977). These indirect pathways include fear acquisition as a result of observing others' fearful reactions (vicarious learning) or of hearing verbal threat information from others (information transmission) about a novel situation.

In earlier empirical research, these indirect fear learning pathways were investigated by exposing children to threatening nonverbal and verbal expressions about novel stimuli (such as dolls), and afterward assessing their attitude as well as emotional, physiological, and behavioral responses towards them (Askew & Field, 2007; Field et al., 2001; Muris & Field, 2010; Reynolds et al., 2014). It has been demonstrated that new fears are readily acquired through social learning alone.

The development of fear and anxiety in childhood often emerges within the family context (Kiel & Kalomiris, 2019). One theoretical model suggests that the way parents talk about, react to, and model emotions (emotion socialization, Eisenberg et al., 1998) is directly related to their children's emotional understanding, expressivity, and regulation. Previous reviews have summarized findings on the role of verbal comments signaling threat on children's fear of novel stimuli. The reviewed evidence suggests that verbal threat information about novel stimuli leads to an increase in child fearful and anxious cognitions, heart rate, and avoidant behavior toward these novel stimuli (Emerson et al., 2019; Muris & Field, 2010; Percy et al., 2016). Thus, there is empirical support for the notion that children can acquire new fears through their parents verbally expressing fear of novel stimuli.

One of the most widely studied risk factors for offspring development of anxiety is parental anxiety. Theoretical models propose that besides passing on genes, parents with an anxiety disorder might also pass on their fears and anxiety via environmental pathways (Gregory & Eley, 2007; Hettema et al., 2001; Hudson & Rapee, 2004; Murray et al., 2009; Percy et al., 2016). Parents with anxiety disorders are more likely to react to novelty with verbal and nonverbal expressions of anxiety. Repeated exposure to parents' anxiety expressions during the confrontation with novel stimuli is a risk factor for the acquisition of fears and anxiety (Murray et al., 2009; Percy et al., 2016). Anxious and nonanxious parents differ in how frequently they verbally communicate threat (Moore et al., 2004; Muris et al., 2010). Furthermore, evidence suggests that the impact of verbal threat information on children's fear beliefs towards novel animals varies between parents with higher and lower trait anxiety. In a sample of 88, 8 to 13‐year‐old children with their parents, they found that parental trait anxiety was associated with giving more threatening narratives about the novel animal, which in turn was associated with increased fear in the offspring of the novel animal (Muris et al., 2010). Parental anxiety might not only affect the amount of anxious signals parents express to their children, but repeated exposure to parents' anxiety might also create a context in which children are sensitized to parent's negative comments (Muris & Field, 2010). Children who grow up in an environment high in conflict or stress might be more receptive to parental negative comments based on learned vigilance to parental cues or increased arousal levels, or might be more affected by these comments (Davies et al., 2006). Nevertheless, until now there is little research on how parental verbal information and parental anxiety interact and possibly shape child acquisition of fear and anxiety (Percy et al., 2016). Establishing whether the link between parental verbal threat information and child fear is stronger for children of parents with higher levels of anxiety might hint at increased salience towards parental cues for these children.

Yet, not every child reacts the same to their parents' expressions of fear about novel situations. The theoretical models on the role of child temperament in the development of child anxiety propose that children with a fearful temperament are more susceptible to environmental stressors such as parental expressions of fear and anxiety (Belsky & Pluess, 2009; Ingram & Luxton, 2005; Nigg, 2006). One well‐studied child temperamental dimension is behavioral inhibition (BI), which has been strongly linked to later anxiety (Clauss & Blackford, 2012). BI is described as a fearful style of reacting to ambiguous stimuli (Fox et al., 2005). Previous studies found that children higher in BI showed increased heart rate and behavioral avoidance towards a novel animal after being exposed to threatening information (Field, 2006; Field & Price‐Evans, 2009). Taken together, parental anxiety and child BI might be possible moderators in the link between parental verbal information about a novel stimulus and offspring fear of that stimulus.

Despite accumulating evidence on the effect of parental verbal communication of threat information on their offspring's fear acquisition in experimental lab designs, research on this link is scarce in the context of novel and potentially threatening situations that parents themselves confront in real‐life settings, such as a pandemic. Due to regulations, such as curfews and lockdowns, families spend a lot of time together at home, which inadvertently means that children are frequently exposed to their parent's emotional reactions and verbal information, including possibly their fear of Covid‐19. An earlier study investigated the link between parents' verbal threat information on child fear acquisition in the context of the swine flu pandemic in a sample of Dutch families (Remmerswaal & Muris, 2011). They reported a positive significant link between parents' communication of threat about the swine flu and offspring fear towards the swine flu (Remmerswaal & Muris, 2011). This study also confirmed that this link was partly accounted for by parents who were more scared of the swine flu being more likely to communicate threatening information. These findings suggest that parental verbal communication as a pathway to child fear acquisition might also hold in the context of a naturally occurring threat, such as a pandemic. Two recent studies investigated the vicarious learning and verbal threat information pathways to Covid‐19 fears. The first one, investigated it in 7 to 19‐year‐old Serbian children (Radanović et al., 2021), using an online survey. They asked parents and their children to report their fear of the virus as well as assessed to what extent parents verbally expressed fear, nonverbally modeled fear, and whether they had Covid‐19. They found that stronger Covid‐19 fear in parents was related to stronger behavioral and verbal fear expressions, which in turn related to their children's increased fear of Covid‐19. The second study examined the role of parental verbal threat information in the parent–child transmission of Covid‐19 fears in 255 families (mainly residing in the United States) with children aged between 5.5 and 17 years (Uy et al., 2022). They found parental verbal fear expressions partially mediated the link between parent and child fear of Covid‐19 in younger children but not adolescents. The study suggests that younger children might be more sensitive to parental verbal threat information. Taken together, the aforementioned studies suggest that the verbal pathway might also hold up in the context of the Covid‐19 pandemic.

In this study, we collected online cross‐sectional survey data about fear of Covid‐19, parent–child communication, parental anxiety, and child temperament from 195, 8 to 18‐year‐old children and their parents in the Netherlands, in the period between June 11, 2020 and May 28, 2021. The aim was twofold. First, the study aimed to extend earlier findings on the link between parent and child fears of Covid‐19 and the mediating role of parental verbal information about Covid‐19 to a Dutch sample. It is important to replicate the studies by Radanović et al. (2021) and Uy et al. (2022) in different countries as fear of Covid is context‐dependent and relates not only to governmental measures but also to cultural differences (Lin et al., 2021). Furthermore, this study is the first to assess parental verbal threat information about Covid‐19 using both child and parent reports. Previous studies reported the benefit of using multiple informants to assess parenting behavior (Renk, 2005). Therefore, including child and parent reports on parental threat communication can provide a more complete picture of whose perspective drives the studied associations. We expected that parents' fear of Covid‐19 would be positively related to their children's fear of Covid‐19 and that this link will be partly mediated by parental verbal threat information.

Second, we explored whether the relationship between parental verbal threat information and children's fear of Covid‐19 is stronger for behaviorally‐inhibited children and children of more anxious parents, compared to children lower in BI and children of parents with lower levels of trait anxiety. While earlier literature discussed the role of child temperament (grounded in the susceptibility theory, Belsky & Pluess, 2009; Ingram & Luxton, 2005; Nigg, 2006) and parental anxiety (Murray et al., 2009; Percy et al., 2016) in the parent–child anxiety transmission, this study will be the first to consider the potential moderating role of parental anxiety and child BI in the context of fear of Covid‐19.

## 2 METHODS

### 2.1 Participants

Participating families were recruited via social media platforms, and via printed flyers and posters, which were distributed in the area of Leiden, The Hague, and Amsterdam. Furthermore, we recruited at high schools in South Holland, by hanging posters, and invitation emails to parents via their secretaries. Inclusion criteria included the participant's proficiency in Dutch or English, as the questionnaires were available in these two languages. Children had to be between 8 and 18‐year‐old and their parents 18 years or older. We only included families in the sample if they currently lived in the Netherlands, and at least one child and one parent filled in the outcome measure on fear of Covid‐19.

The Fear of Covid‐19 questionnaire (FCQ) was filled in by 280 parents and 225 children. Data from 25 children were excluded as they did not have the accompanying parent data. The data of additional five children and accompanied parent data were deleted because the children fell outside of the age range. Eighty‐two of the parent responses were deleted, as there were no linked child responses. The final sample consisted of 195 Dutch parent–child dyads. Demographic information about the final sample can be found in Table 1.

**Table 1:** Sample characteristics

Parents *N*	193	Children *N*	195
Age Mothers *M* (*SD*)	47.26 (5.86)	Age *M* (*SD*)	14.23 (2.99)
Age Fathers *M* (*SD*)	49.58 (6.33)	Girls *N (%)*	113 (58)
Mothers *N (%)*	146 (76)	Birthplace Netherlands *%*	95.3
Education level %		School	
Primary professional education	1.56	Primary school %	28.42
(Higher) secondary education	7.81	Secondary education %	56.84
Secondary scientific education	1.56	Other %	14.74
Secondary professional education	18.75		
Higher professional education	36.98		
Higher scientific education	33.33		
Work %			
Part‐time	47.15		
Full‐time	40.41		
Sick leave	2.07		
No work	5.70		
Other	4.15		

Abbreviations: *M*, Mean; *N*, sample size; SD, standard deviation.

### 2.2 Procedure

Participants were introduced to the purposes of the study and gave their online informed consent before filling in the questionnaires. The children had to fill in the questionnaires independently from their parents. The questionnaires took approximately 20 min to complete. Families who joined the study could take part in a raffle, in which 1 in every 50 participating families would receive 50 euros. The study was approved by the Leiden University Psychology Ethics Committee (#2020‐05‐15‐Aktar‐V1‐2456).

### 2.3 Materials

#### 2.3.1 Fear of Covid‐19

Fear of Covid‐19 was measured with the FCQ, which is an adaptation of the Fear of Swine Flu Questionnaire (FSFQ; Remmerswaal and Muris [2011]). The FSFQ consists of 12 items with a 4‐point scale (1 = not true to 4 = very true). We created the FCQ by replacing the term swine flu with Covid‐19 and adding two items. The two added items in the parent/child version are “Do you think something bad will happen to your child/parents if they had Covid‐19?” and “Would you be scared if your child/parent has Covid‐19?” A higher mean item score represents a higher level of fear of Covid‐19. The reliability of the adapted questionnaire in the current sample is 0.78 for the children and 0.85 for the parents.

#### 2.3.2 Parental communication of threat information

We assessed the quantity of parental communication of threat about Covid‐19 with an adapted version of the Source of Information about the Swine Flu Scale (SISFS, Remmerswaal & Muris, 2011). The SISFS assesses how children acquire information about the swine flu from various sources. The original scale consists of 10 items on a 4‐point scale. For the present study, we adapted these items by exchanging the term swine flu with Covid‐19. Due to our focus on parent communication, we used the four items relating to parent verbal communication of threat. In these four items, parents are asked about the specific information they share with their children regarding Covid‐19, whereas children are asked about the information they receive from their parents about the virus. A final mean item score of the combined child and parent reports was computed. The higher the mean, the more frequently the parent exchanged threat‐related information about Covid‐19 with their child. The reliability of the adapted questionnaire in the current sample is 0.84 for the combined variable.

#### 2.3.3 BI

Child BI was measured with the Behavioral Inhibition Scale (BIS) from Carver and White's (1994) BIS/BAS scales. This subscale consists of seven items (i.e., “I feel worried when I think I have done poorly at something”), on a 4‐point scale (1 = strongly disagree to 4 = strongly agree). The higher the final mean score, the more behaviorally inhibited the child is. The reliability of the questionnaire in the current sample is 0.76.

#### 2.3.4 Parental anxiety

Parents reported their anxiety in the adult version of Screen for Child Anxiety Related Disorders SCARED‐A (Bögels & Melick, 2004). The SCARED‐A consists of 71 items on a 3‐point Likert scale (0 = almost never to 2 = often). Higher scores on this scale indicate a higher level of parent anxiety. The reliability of the questionnaire in the current sample is 0.93.

### 2.4 Statistical analyses

First, the data was manually inspected to check for erroneous data. Parents that were excluded based on missing child responses did not differ based on any of the study variables (such as parent report of fear of Covid, frequency of verbal threat information or family variables, such as parental gender or child gender), all *p* > .13. Then we checked means and standard deviations of all variables, outliers, and the normality of distributions. To simultaneously test the associations between child fear of Covid‐19, parent fear of Covid‐19, and parental verbal threat information, a structural equation model was computed in R, using the lavaan package. To test whether the link between parent fear of Covid‐19 on child fear of Covid‐19 was mediated by parental verbal information, we assessed the parameter estimate of the indirect effect of parental verbal information. Based on the findings regarding age in Uy et al. (2022) study, we added child age as a covariate in all analyses. Additionally, we explored whether the link between parental verbal threat information on child fear of Covid‐19 was stronger for children higher in BI, and children with parents higher in anxiety symptoms. Predictors were centered on the group mean, and two interaction variables between (1) parent trait anxiety and parental threat communication and (2) child BI with parental threat communication were created. The model parameters were calculated with a Maximum Likelihood estimation (Rosseel, 2012), and the hypotheses were tested bi‐directionally, reporting bootstrapped (1000 iterations) 95% confidence levels (CIs). Full Information Maximum Likelihood was used to handle missing values.

## 3 RESULTS

### 3.1 Preliminary analyses

Three outlying scores (one on child fear of Covid‐19 and two on parental anxiety) with *z*‐values larger than 3.29 or smaller than −3.29 were found. Analyses were done with and without outliers. Results did not significantly differ at *α* level of .05. Therefore, the outlying scores were retained in the final analyses (Tabachnick & Fidell, 2013). Means, standard deviations and correlations between the study variables are presented in Table 2. The descriptives and correlations between the separate mother and father scores of the study variables can be found in the Supporting Information: Table S1.

**Table 2:** Descriptives, reliabilities, and intercorrelations of study variables

	*N*	*M*	*SD*	1	2	3	4	5
1. Parent verbal Info	195	2.65	0.62	‐	.44***	.55***	.01	.13
2. Child fear of Covid	195	2.15	0.45		‐	.43***	−.22**	.16*
3. Parent fear of Covid	195	2.16	0.45			‐	.02	.25**
4. Child BI	192	2.26	0.61				‐	−.08
5. Parent anxiety	171	0.32	0.21					‐

Abbreviations: BI, behavioral inhibition; *M*, mean; *N*, sample size; SD, standard deviation.

* *p* < .05

** *p* < .01

*** *p* < .001.

### 3.2 Main analyses

**Figure 1:**
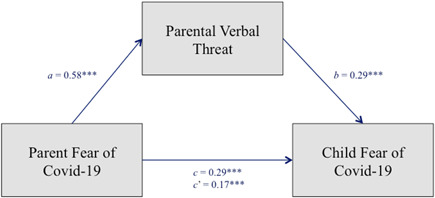
Path Model with parental verbal information as mediator between parents' and children's fear of Covid‐19. Children's age was used as covariate but is not depicted. Statistics are standardized regression coefficients. *c*,direct effect, *c'*, indirect effect. [Color figure can be viewed at wileyonlinelibrary.com]

In the first structural equation model the associations between child fear of Covid‐19, parent fear of Covid‐19, and parental verbal threat information were simultaneously tested (see Table 3, Figure 1). Parent fear of Covid‐19 was positively associated with child fear of Covid‐19, *β* = .29, *SE* = 0.08, CI (0.13, 0.46), *p* < .001. Furthermore, the more scared parents were of Covid‐19, the higher the frequency of verbal threat information about Covid‐19 towards their children, *β* = .58, *SE* = 0.07, CI (0.46, 0.72), *p* < .001. The covariate child age was not significant (*β* = .06, *SE =* 0.02, CI [−0.03, 0.06], *p* = .38). Additionally, parents' communication of threatening information about Covid‐19 to their child was positively related to child fear of Covid‐19, *β* = .29, *SE* = 0.09, CI (0.13, 0.49), *p* < .001. Child age was not related to parents' communication of threatening information, *β* = −0.07, *SE* 0.02, CI (−0.06, 0.01), *p* = .21. The indirect effect of parental communication of verbal threat information about Covid‐19 in the link between parent fear of Covid‐19 and child fear of Covid‐19 was significant, *β* = .17, *SE* = 0.06, CI (0.08, 0.31), *p* < .01. Given that parental verbal threat information was a combined variable between parent and child reports on parental threat communication, we explored whether these results differ depending on the reporter, by repeating the analyses with parent report of parental verbal threat information, as well as child report of parental threat information as parallel mediators in the same model (see additional analyses in Supporting Information: Figure S1). Findings from the parallel mediation indicate that the link between parent and child fear of Covid‐19 is partly explained by the children's but not parents' reports of parental verbal threat information.

**Table 3:** Mediation analysis

						95% CI		
Direct effect			*β*	*SE*	*z*	*p* Value	Lower	Upper
Parent fear of Covid	Child fear of Covid		0.29	0.08	3.66	<.001	0.13	0.46
Indirect effect								
Parent fear Covid	Parent verbal info	Child fear Covid	0.17	0.06	3.07	<.01	0.07	0.29

*Note*: Delta method standard errors, bias‐corrected percentile bootstrap confidence intervals.

Concerning the moderating roles of parental anxiety and child BI, the model revealed that the relationship between parental verbal threat information and child fear of Covid‐19 was not moderated by child temperament or parental anxiety levels. In other words, we found no support to the idea that the association between parental verbal threat information and child fear was not stronger for children with higher levels of BI (*β* = .00, *SE* = 0.07, CI [−0.15, 0.13], *p* = .996), or children of parents with higher levels of anxiety (*β* = .03, *SE* = 0.09, CI [−0.16, 0.18], *p* = .71). Child age was not significantly linked to child fear of Covid‐19 in either moderation model (*p* > .26).

## 4 DISCUSSION

The present study investigated the link between parents and children's fear of Covid‐19, and whether this link can be at least partially accounted for by parental verbal threat information. Additionally, we explored whether the relationship between parental verbal threat information and children's fear of Covid‐19 is stronger for children higher in BI and children of parents with higher levels of trait anxiety. In line with our hypotheses, parents who were more scared of Covid‐19 have children who were also more scared of Covid‐19. Furthermore, parents who were more scared of Covid‐19 provided more frequent negative comments about the virus to their children. Parental negative comments, in turn, were related to higher levels of Covid‐19 fears in their children. These findings are in line with earlier literature, which reported that parents' fear of a novel stimulus was related to higher levels of behavioral and verbal fear expressions, which in turn related to their children's increased fear of that novel stimulus (Muris et al., 2010; Radanović et al., 2021; Remmerswaal & Muris, 2011; Uy et al., 2022). Like Radanović et al. (2021), we also found parental verbal information to partially mediate the link between parent and child fear of Covid‐19. Lastly, we did not find age to relate to child fear of Covid‐19 in our models. This is in line with earlier work, which also did not find age to be a confounding factor in the verbal transmission pathway (Radanović et al., 2021). However, they did find children's age to be linked to children's fear of Covid in the parental modeling pathway as well as in the nonfamily information pathway (Radanović et al., 2021). The influence of children's age might be stronger in the transmission of fears via other learning pathways than parental verbal fear transmission. More research is needed to disentangle in which developmental phase various fear transmission pathways relating to Covid‐19 are especially salient.

Interestingly, based on our exploratory analyses, our model combining child‐reported and parent‐reported scores of parental verbal threat information was driven by the child's perspective on parental threat information. While the frequency of parental information reported by children partially mediated the link between parent and child fear of Covid‐19 in the model, parent‐reported scores of verbal threat information did not, as it was not linked to child fear of Covid‐19. This discrepancy might be due to parents being less aware of their communications about Covid‐19 to their child, the child's sensitivity to or inflated interpretation of parental verbal threat information, especially when they are already scared of Covid‐19. These findings highlight the importance to consider possible reporter bias when investigating parent–child communication or interactions using self‐report.

In our study, parental verbal information accounted for only 8% of the variance in children's fear of Covid‐19, when taking into account parent's fear of Covid‐19 and child age. Thus, other factors play an important role in children's fear acquisition. One of these factors concerns the nonverbal pathways to social learning. Parents' fear of Covid‐19 was earlier found to be related to parental modeling of their fear of the virus and to children's fear of the virus (Radanović et al., 2021). However, parental fear modeling did not mediate the relationship between parent and child fear of Covid‐19, suggesting that nonverbal parental transmission cannot by itself explain the family fears of Covid‐19. Another factor that may play a role in the overlap in parent and child fear of Covid‐19 is their shared environment. If parents and children watch the same news or talk to family friends who voice or express their fears about the virus in front of both the parents and their children, this might shape their fear acquisitions similarly. Radanović et al. (2021) found that verbal information from other sources (such as teachers, peers, TV, or the Internet) significantly contributed to children's fear of the virus. However, they did not investigate whether parents and children share the same information sources and their impact on parent and child fears of the virus. Lastly, in exploratory analyses, we checked whether children who had direct exposure to the virus were more scared of Covid‐19. In line with Uy et al. (2022), we did not find child's exposure to the virus to be linked to children's fear of Covid‐19, which suggests that it is not likely a confounding factor in the parent–child transmission of Covid‐19 fears. Nevertheless, subsequent research should expand our study by investigating the influence of other information sources or direct exposure to the virus on child fear acquisition towards Covid‐19.

We also assessed parental anxiety and child BI as moderators in the link between parental verbal threat information and child fear of Covid‐19. No support was found for the idea that the link between parental verbal threat information and children's fear of Covid‐19 was stronger for children of parents with higher levels of anxiety. It is important to note that in our community sample parents reported very few anxiety symptoms. The lack of moderation might be explained by the little variance in parental anxiety. Future studies could also investigate parental stress, which is more represented in community samples and is a risk factor for anxiety development (Pêgo et al., 2009). Compared to anxiety measures which often focus on physical manifestations of anxiety (such as trouble breathing and trembling), stress scales focus less on physical symptoms, and more on agitation and lower frustration tolerance (see Lovibond & Lovibond, 1995). Parental stress has increased since the onset of the Covid‐19 pandemic (Lucassen et al., 2021). Similar to anxiety, parental stress might diminish parents' emotion regulation skills or make them express their fears more intensely (Havighurst & Kehoe, 2017). Given that stress is increased during this stressful period, and might inhibit parents' ability to regulate their own fears, highly stressed parents might be expressing their Covid‐19 fears more frequently or intensely compared to less stressed parents. Hence, future studies could look into increased parental stress as a possible moderator in the link between parental verbal threat information and child fear of Covid‐19. While parental anxiety was not associated with the amount of verbal threat information provided to their children, it was linked to parental and child fear of the virus. This might indicate that broader anxiety disposition in parents might not influence how they react to this novel virus themselves but might still impact their own and their children's development of fear of the virus. Future research could investigate other mechanisms in which parental anxiety might influence the development of child fear of Covid‐19.

Lastly, we also did not find child temperament, specifically child BI, to moderate the relationship between parental verbal information and child fear of Covid‐19. This contrasts the findings of previous studies that the relationship between verbal threat information on child fear towards novel animals is stronger for behaviorally inhibited children (Field & Price‐Evans, 2009; Field, 2006). Note, however, that the previous studies assessing this link have measured child fear towards novel animals in a laboratory setting, being exposed to information of only the experimenter or the parent, before their fears towards the novel animals were assessed. In contrast, our study started after the Covid‐19 outbreak had already been named a pandemic, which means that we assessed children's fear of Covid‐19 after they had been informed about this virus from various sources.

The current study is limited in that it remains unknown whether children's first “encounter” or information they received about the virus was from their parents, or whether they have already formed (fear) beliefs about the virus beforehand. Since BI relates to fear of *novel* stimuli, it is possible that behaviorally‐inhibited children are not more strongly affected by parental verbal information, because they have received less threatening information from various other sources beforehand, and the virus was not a novel stimulus anymore. Furthermore, the BI scores in our sample were overall lower than the averages reported in nonclinical samples of children (Muris et al., 2005). The little variance in children's BI may have also limited our ability to find a moderating role of BI in the link between parental verbal threat information on child fear of Covid‐19. Lastly, against expectations, there was a weak negative link between children's BI and their fear of Covid‐19: lower levels of BI were related to stronger Covid‐19 fears. One possible explanation is that children with lower levels of BI may have exposed themselves more often to situations that would potentially directly expose them to the virus, whereas children high in BI possibly stayed more at home, avoided the news, and exposed themselves less to the virus or others expressing fears. This avoidant style may have helped the regulation of fears in this context, and dampened the fear‐inducing impact of parent verbal threat information about the virus. Taken together, we found no direct support for the idea that parental anxiety or child BI are risk factors in the link between parental verbal threat information and children's fear of Covid‐19. Future research should continue the quest to find out which children might be at increased risk to develop fear towards Covid‐19 after being exposed to parents' comments.

Our study is the first to investigate Rachman's verbal fear acquisition pathway in the context of Covid‐19 using a Dutch sample, while also assessing possible risk factors that might strengthen children's fear learning. Furthermore, in contrast to previous work, this study assesses parental verbal threat information about Covid‐19 with both child and parent reports. Nevertheless, the following limitations should be taken into account. First, reported associations are correlational, meaning we cannot infer causality in this cross‐sectional design. Second, the study was conducted over the span of a year, and the Covid‐19 infection rates and governmental measures to combat the infection rates differed during the various stages of the pandemic. Future research that assesses Covid‐19 fears at multiple time points could assess fluctuations of parental and child fear of Covid‐19 and the influence of parental comments while taking the severity of the pandemic into account. Third, the current parent sample consisted mainly of mothers (76%). A previous study found mothers to voice more Covid‐19 concerns and display more safety behaviors than fathers (Lauri Korajlija & Jokic‐Begic, 2020). In our sample, mothers report more Covid‐19 fears and more frequent communication of their fears than fathers (see Supporting Information: Table S1). Due to the overrepresentation of mothers in our study, the generalizability of the results to fathers is limited, and future research should expand our findings by incorporating both parents. Lastly, this study should be interpreted in the context that at the point of data collection Covid‐19 fears were adaptive given the novelty of the virus, the absence of vaccines or medication, and therefore possibly high severity of threat. Additionally, our sample contains parents with overall low levels of anxiety. Hence, no conclusion can be drawn to the transmission of maladaptive fears/clinical fears/Covid‐19 anxiety. Despite these limitations, the current study contributes to our knowledge of Rachman's social fear learning model, by highlighting the role of parental communication in children's fear acquisition in a typically developing sample of 8 to 18‐year‐old and their parents during the Covid‐19 pandemic. While parental anxiety and child BI did not moderate the relationship between parental verbal threat information and child fear of Covid‐19, the link of parental anxiety to child fear of Covid‐19 warrants further investigation.

## CONFLICT OF INTEREST

The authors declare no conflict of interest.

## ETHICS STATEMENT

The study was approved by the Leiden University Psychology Ethics Committee (#2020‐05‐15‐E. Aktar‐V1‐2456).

## Supporting information

Supporting information.

## Data Availability

The data that support the findings of this study are available from the corresponding author upon reasonable request. All raw data and materials will be stored online within 1 month after publication to DataverseNL, available upon request from the corresponding author.
