# Supplementary material for: “Covid‐19 is dangerous”: The role of parental verbal threat information on children's fear of Covid‐19
Source: J Adolesc. 2022 Oct 25;95(1):147–56. doi: 10.1002/jad.12105 (PMC9874763; doi:10.1002/jad.12105)
Supplement: Supplementary file 1 — Supporting information. [file JAD-95-147-s001.docx]

**Supplementary Materials**

Table S1.

|  | *N* | *r* | *M* | *SD* | 1 | 2 | 3 | 4 | 5 | 6 |
| --- | --- | --- | --- | --- | --- | --- | --- | --- | --- | --- |
| 1. Mother Fear of Covid | 143 | .84 | 2.19 | 0.48 | - | .49* | .62*** | .40 | .23** | .03 |
| 2. Father Fear of Covid | 50 | .74 | 2.10 | 0.37 |  | - | .44* | .53*** | -.10 | .24 |
| 3. Mother Verbal Info | 140 | .81 | 2.69 | 0.70 |  |  | - | .56** | .13 | -.17 |
| 4. Father Verbal Info | 50 | .81 | 2.60 | 0.74 |  |  |  | - | .24 | -.16 |
| 5. Mother Anxiety | 142 | .94 | 0.34 | 0.22 |  |  |  |  | - | .08 |
| 6. Father Anxiety | 50 | .94 | 0.25 | 0.20 |  |  |  |  |  | - |

Descriptive information and correlations between mother and father variables

*Notes. N* = sample size, *r* = reliability, *M* = Mean, *SD* = Standard deviation, * = *p* < .05, ** *p* < .01, *** = *p* < .001.


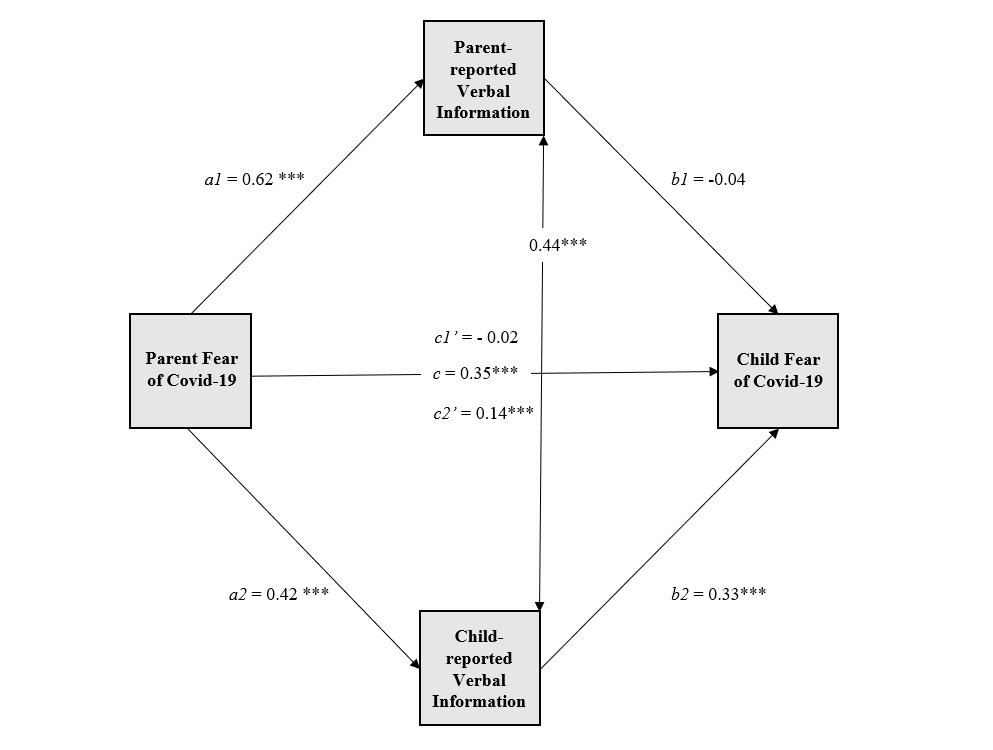


Figure S1. Path Model with parent and child-reported parental verbal information as parallel mediators between parents’ and children’s fear of Covid-19.

Notes. *c*= direct effect, *c1’*= indirect effect of parent-reported verbal information, *c2’*= indirect effect of child-reported verbal information. Children’s age was included as covariate but is not depicted. Statistics are standardized regression coefficients.
